# Effect of MMR Vaccination to Mitigate Severe Sequelae Associated With COVID-19: Challenges and Lessons Learned

**DOI:** 10.18103/mra.v11i2.3598

**Published:** 2023-02

**Authors:** Mairi C. Noverr, Junko Yano, Michael E. Hagensee, Hui-Yi Lin, Mary C. Meyaski, Erin Meyaski, Jennifer Cameron, Judd Shellito, Amber Trauth, Paul L. Fidel

**Affiliations:** 1 Department of Microbiology and Immunology, Tulane University School of Medicine; 2 Center of Excellence in Oral and Craniofacial Biology, LSU Health School of Dentistry; 3 Section of Infectious Diseases, Department of Medicine, LSU Health New Orleans; 4 Biostatistics Program, LSU Health School of Public Health; 5 Clinical and Translational Research Center, LSU Health New Orleans; 6 Department of Microbiology, Immunology, and Parasitology, LSU Health New Orleans; 7 Section of Pulmonary Medicine, Department of Medicine, LSU Health New Orleans

## Abstract

Mortality in COVID-19 cases was strongly associated with progressive lung inflammation and eventual sepsis. There is mounting evidence that live attenuated vaccines commonly administered during childhood, also provide beneficial non-specific immune effects, including reduced mortality and hospitalization due to unrelated infections. It has been proposed that live attenuated vaccine-associated non-specific effects are a result of inducing trained innate immunity to function more effectively against broader infections. In support of this, our laboratory has reported that immunization with a live attenuated fungal strain induces a novel form of trained innate immunity which provides protection against various inducers of sepsis in mice via myeloid-derived suppressor cells. Accordingly, we initiated a randomized control clinical trial with the live attenuated Measles, Mumps, Rubella (MMR) vaccine in healthcare workers in the greater New Orleans area aimed at preventing/reducing severe lung inflammation/sepsis associated with COVID-19 (ClinicalTrials.gov Identifier: NCT04475081). Included was an outcome to evaluate the myeloid-derived suppressor cell populations in blood between those administered the MMR vaccine vs placebo. The unanticipated emergency approval of several COVID-19 vaccines in the midst of the MMR clinical trials eliminated the ability to examine effects of the MMR vaccine on COVID-19-related health status. Unfortunately, we were also unable to show any impact of the MMR vaccine on peripheral blood myeloid-derived suppressor cells due to several inherent limitations (low percentages of blood leukocytes, small sample size), that also included a collaboration with a similar trial (CROWN CORONATION; ClinicalTrials.gov Identifier: NCT04333732) in St. Louis, MO. In contrast, monitoring the COVID-19 vaccine response in trial participants revealed that high COVID-19 antibody titers occurred more often in those who received the MMR vaccine vs placebo. While the trial was largely inconclusive, lessons learned from addressing several trial-associated challenges may aid future studies that test the non-specific beneficial immune effects of live attenuated vaccines.

## Introduction

Epidemiological studies on the use of live attenuated vaccines (LAVs) have reported beneficial non-specific effects, including reduced mortality and hospitalization due to unrelated infections.^1–3^ For example, MMR vaccination is associated with significantly reduced risk of hospitalization due to unrelated infectious diseases, particularly respiratory infections.^4^ These non-specific effects are hypothesized to be a result of induction of trained innate immunity (TII). Mechanistically, live vaccines have been shown to traffic to the bone marrow where they can train leukocyte precursors to function more effectively against broader infectious insults.^5,6^ Work from our laboratory demonstrated that immunization with a live attenuated fungal strain induces trained innate protection against various inducers of sepsis. Unlike other forms of TII, we discovered that protection against sepsis is mediated by long-lived Gr-1+ myeloid-derived suppressor cells (MDSCs).^6–9^ In other experimental sepsis models, MDSCs have been shown to inhibit pathological inflammation and prevent mortality (reviewed in ^10^). Therefore, we have proposed the term trained tolerogenic immunity ^10^ to discriminate this form of TII from the previously identified trained immunity involving enhanced inflammatory responses. ^11–13^

Mortality due to COVID-19 is associated with severe acute respiratory distress syndrome (ARDS) and sepsis, characterized by high SOFA (sequential organ failure assessment) score, an indicator of sepsis.^14^ Prior to the 3^rd^ and 4^th^ wave of the COVID-19 pandemic (October 2020), the overall death rate among all COVID-19 patients in the US was 3%. This rate increased to 14–24% if hospitalized and >40% if additionally admitted to the ICU. In response to the COVID pandemic, we postulated that the live attenuated MMR vaccine administered to adults could induce trained MDSCs as a stop-gap measure to inhibit the pathological inflammation/sepsis associated with COVID-19. We outlined this strategy in a well-received and widely discussed commentary^15^ and subsequently initiated a randomized control clinical trial (MMR vs placebo) in eligible healthcare workers (HCWs) in the greater New Orleans area (ClinicalTrials.gov Identifier: NCT04475081) through the Louisiana State University Health Sciences Center. The trial was ultimately terminated early because the COVID vaccines became widely available a few months after enrollment began (Dec. 2020). However, we completed the trial in all those enrolled (n=34) and entered into a collaboration with colleagues at the Washington University School of Medicine in St. Louis who were conducting a similar, but more global trial evaluating the effects of the MMR vaccine in HCWs in nine countries (CROWN CORONATION; ClinicalTrials.gov Identifier: NCT04333732). A sub study within the CROWN CORONATION trial collected blood samples from participants in St. Louis, MO post-MMR/placebo and COVID-19 vaccination. Cytokine and chemokine responses to SARS-CoV-2 and other stimuli were measured by the study team in St. Louis (ClinicalTrials.gov Identifier: NCT04646239), and a portion of the samples were shipped for MDSC analysis as per the protocol of the LSU study team. ^16^

It is important to point out that other investigative groups have initiated similar clinical trials using LAV for the purpose of inducing cells trained for non-specific immune enhancement ^17^, including against SARS-CoV-2.^18–20^ However, our overarching hypothesis is that administration of the live attenuated MMR vaccine would induce the trained innate tolerogenic MDSCs that would mitigate pathology-inducing inflammation/sepsis. Here we summarize the results of the clinical trial relative to the MDSCs and serology for both the MMR and COVID vaccine-related antibodies. We also outline a number of challenges faced during the trial and lessons learned that may apply to similar trials in the future.

## Materials and Methods

### Clinical Design.

Eligible healthcare workers (HCWs) in the greater New Orleans area (n=34) meeting eligibility criteria were enrolled into a 12-month study by the Louisiana State University Health Sciences Center (LSUHSC) Clinical & Translational Research Center (CTRC) affiliated with the larger statewide Louisiana Clinical and Translational Research Science (LA CaTS) Center. The study was approved by the Institutional Review Board (IRB) associated with Louisiana State University Health – New Orleans. The subjects were blindly randomized to receive the live attenuated M-M-R^®^ II vaccine or placebo (sterile saline) via subcutaneous injection in the arm at a baseline visit following informed consent. Subjects were recruited from local hospitals throughout the greater New Orleans area. Subject consenting, interviewing, vaccine administration and biospecimen collection was performed by the CTRC staff, under full Personal Protective Equipment (PPE) protection. Following informed consent, subjects were asked to complete the Baseline Demographic & History Questionnaire disclosing their demographic information, employment, medication, vaccination, and medical history. Specifically, the medical history placed emphasis on the presence of diabetes, hypertension, heart disease, and their treatments/medications. Subjects then had their height, weight, body mass index (BMI), vital signs, and pulse oximetry measured. Female subjects of childbearing potential were given a urine-based pregnancy test (Instant-View Pregnancy Urine Cassette Test, Alfa Scientific Designs Inc). A total of 20cc of blood was collected along with a nasopharyngeal swab for baseline laboratory analyses (serology, viral RNA, flow cytometry). In total, 34 participants were enrolled; 15 in the placebo group and 19 in the MMR group. The demographics were as follows: The median age was 52 yrs (range 25–81 yrs) with 70% female. Ethnicity consisted of 73% white, 9% black, 9% Latino and 9% other with excellent matching between the respective arms.

Repeat biosampling occurred on days 14, 30, and 60 post-injection. A subset of subjects also had blood sampling at 6–8 months post-vaccination to match the time point of a collaboration with Washington University School of Medicine in St. Louis, MO (see details below). A portion of the whole blood samples (10cc) was shipped overnight on the day of collection to a flow cytometry testing facility (Flow Contract Site Laboratory, Bothell, WA). The sera from the remaining blood sample was aliquoted and stored at −80°C. At each follow-up visit, anthropometric measurements, vital signs measurement, and symptom assessment for the presence of symptoms related to SARS-CoV-2 infection (fever, headache, myalgia, cough, loss of taste or smell, breathing problems), general well-being (i.e., pain, dental concerns, sleep patterns, general stress level, fatigue), and any changes in medications, medical status, and employment were also collected utilizing the Follow-up Symptom & History Questionnaire. Telephone follow-up calls utilizing the Follow-up questionnaire were made on a monthly basis between the 60-day follow-up visit and the 12-month endpoint visit. If a subject developed symptoms potentially associated with SARS-CoV-2 infection at any point during the 12-month study period, he/she was seen in the clinic by the Infectious Disease (ID) Co-investigator (MEH), and a repeat collection of blood and nasopharyngeal biospecimens were performed for analysis, and the subject was asked to complete the Follow-up questionnaire. Subjects were asked to report the development of any potential COVID-19-related symptoms or positive SARS-CoV-2 infection testing outside of the study, as well as any symptoms potentially related to MMR vaccination. The COVID-19 vaccines (when available) were not denied to any enrollee and COVID-19-vaccinated individuals were accepted into the trial based on outcome measures that could still theoretically be met.

Primary outcome measures were peripheral blood monocytic MDSCs (M-MDSC) and/or granulocytic MDSCs (G-MDSC) determined by flow cytometry from whole blood samples at baseline and again at 14, 30, and 60 days post-injection of the MMR vaccine or placebo. Other outcome measures included antibodies to measles and/or mumps post-MMR vaccination as a confirmation for a positive response to the vaccine, and SARS-CoV-2 RNA testing at baseline, 14, 30, and 60 days post-vaccination, and at any point over the 12-month period that symptoms arose. Secondary outcome measures were detection of SARS-CoV-2 antibodies (seropositive) and any evidence of infection, sepsis/lung inflammation, ICU/ventilator usage, in-patient health related co-morbidities and self-reporting mental status (such as general fatigue/stress level) over the 12-month period post-vaccination, predominantly via self-reporting utilizing the Follow-up Symptom & History Questionnaire.

The study in HCW was initiated in Sept 2020 with an expected sample size of 50 (n=25/arm). The trial began with steady but slower than expected enrollment. However, in December 2020 with ~30 subjects enrolled, novel COVID-19 mRNA vaccines were given emergency FDA approval (Pfizer or Moderna) for HCW and first responders. While those with detectable antibodies to SARS-CoV-2 (evidence of previous exposure/infection) were initially excluded from our trial, we allowed COVID-19 vaccinated individuals to enroll despite the fact that the secondary outcome of COVID-19 related health status would be compromised. We also retained individuals already enrolled that subsequently chose to receive a COVID-19 vaccine. To improve the potential to monitor secondary outcomes of COVID-19-related health status, we opened enrollment to the public. Although we enrolled several more subjects, interest waned as COVID vaccines became more widely approved and available to the general public. Thus, we were unable to thoroughly evaluate the outcome of the MMR vaccine on COVID-related health issues.

In addition to the LSUHSC cohort, our clinical trial group initiated a collaboration with investigators at Washington University School of Medicine, St. Louis, MO who were conducting a similar clinical trial in HCWs in several countries, entitled CROWN CORONATION (ClinicalTrials.gov Identifier: NCT04333732). While blood samples were not collected at baseline, a sub study was initiated to collect 20cc of blood from those administered the placebo (n=42) or MMR vaccine (n=46) in the St. Louis cohort at 6–8 months after injection of the MMR or placebo injection (NCT04646239). A portion of the blood sample (10cc) was shipped overnight to the Flow Contract Site Laboratory for MDSC analysis as per LSU protocol. All the enrolled subjects were also COVID-19 vaccinated during the period of the sub study. The demographics of the St. Louis cohort were as follows: The median age of those enrolled was 41 yrs (range 25–71 yrs) with 66% female. The ethnicity included 84% white, 3% Latino, 6% Asian, and 7% other with excellent matching between the arms.

### Laboratory Design

Blood samples were used for analysis of leukocyte populations, and detection of SARS-CoV-2, measles and mumps antibodies. Nasopharyngeal sampling was used to detect SARS-CoV-2 RNA. Urine samples were used to test for pregnancy.

#### Peripheral Myeloid-Derived Suppressor Cell Analysis:

M-MDSCs (CD33^high^CD14^high^HLA-DR^dim^) and G-MDSC (CD33^high^CD66b^high^HLA-DR^dim^) populations were analyzed in the whole blood sample. For the MMR group, samples were evaluated at baseline, 14, 30, and 60 days post-vaccination. For the placebo group, although blood was drawn at the same time points, only the baseline and 30-day samples were evaluated for MDSC populations. In some participants (n=16) an additional sample was collected at 6–8 months to match the sampling protocol of the colleagues in St. Louis for the purpose of MDSC analyses.

#### M/M/R Serological Analysis:

Approximately 10 ml of whole blood was collected into a Vacutainer tube containing clot activator (BD Biosciences). Blood sera were obtained by centrifugation at 1000x *g* for 10 min and analyzed for measles and mumps IgG titers by ELISA (Alpha Diagnostic Intl.; San Antonio, TX) at baseline and several follow-up visits as a measure of vaccine efficacy.

#### SARS-CoV-2 Polymerase Chain Reaction Detection:

Nasal swabs were tested for SARS-CoV-2 RNA using RT-qPCR Rapid Detection Kit according to manufacturer’s instructions (MyBioSource; San Diego, CA).

#### SARS-CoV-2 Serological Analysis:

Serum samples were tested for the presence of antibodies against SARS-CoV-2 nucleocapsid and Spike protein (receptor binding domain, RBD) by in-house ELISA assays for evidence of clinical infection or COVID-19 vaccination, respectively. RBD (Arg319-Phe541) or nucleocapsid, (0.05 micrograms per well) of wild type SARS-COV-2 (Ray Biotech, Peachtree Corners, GA) were placed on Immunolon 2 plates (Thermo-Fisher, USA) in 0.9 M Sodium carbonate buffer (pH 9.5), blocked (Tris, 10mM, NaCl 0.15M, Tween 0.5%m goat serum 10%) and sera added at 2-fold dilutions starting at 1/10, detected with goat-anti-human IgG (H and L) antibody (Invitrogen). Negative controls were derived from archived serum samples collected prior to 2015. End-point dilution titers were defined as the lowest dilution of sera that produced a signal greater than 3-standard deviations over the average of the negative controls.

### Statistical analysis

Subject data for MDSC populations, measles and mumps virus antibody titers, and SARS-CoV-2 spike protein antibody titers were recorded using means and 95% confidence intervals (CIs) for the two study groups (placebo and MMR) at each time point. in M-MDSC and G-MDSC populations between the placebo and the MMR groups at each time-point were analyzed using the Student’s t-test. The differences in the percentages of MDSC populations between baseline and the subsequent time points post-vaccination within a study group were analyzed using the paired Student’s t-test. Changes in measles and mumps virus antibody titers between a specific time-point and baseline for a study group were calculated using log2 fold changes and data analyzed using the paired Student’s t-test. In addition, comparisons in measles and mumps virus antibody titers between the two study groups at any one time point were analyzed using the Student’s t-test. To follow the normality assumption, the log2-transformed SARS-CoV-2 spike protein titers were used for further analyses. Comparisons in SARS-CoV-2 spike protein antibody titers between the MMR and placebo groups were analyzed using the Student’s t-test. In addition, the high titer status of the SARS-CoV-2 spike protein (Log2-transformed titers >15.3 or raw titers >40,000) between the two study groups was evaluated using Fisher’s exact test.

## Results

**Figure 1.**
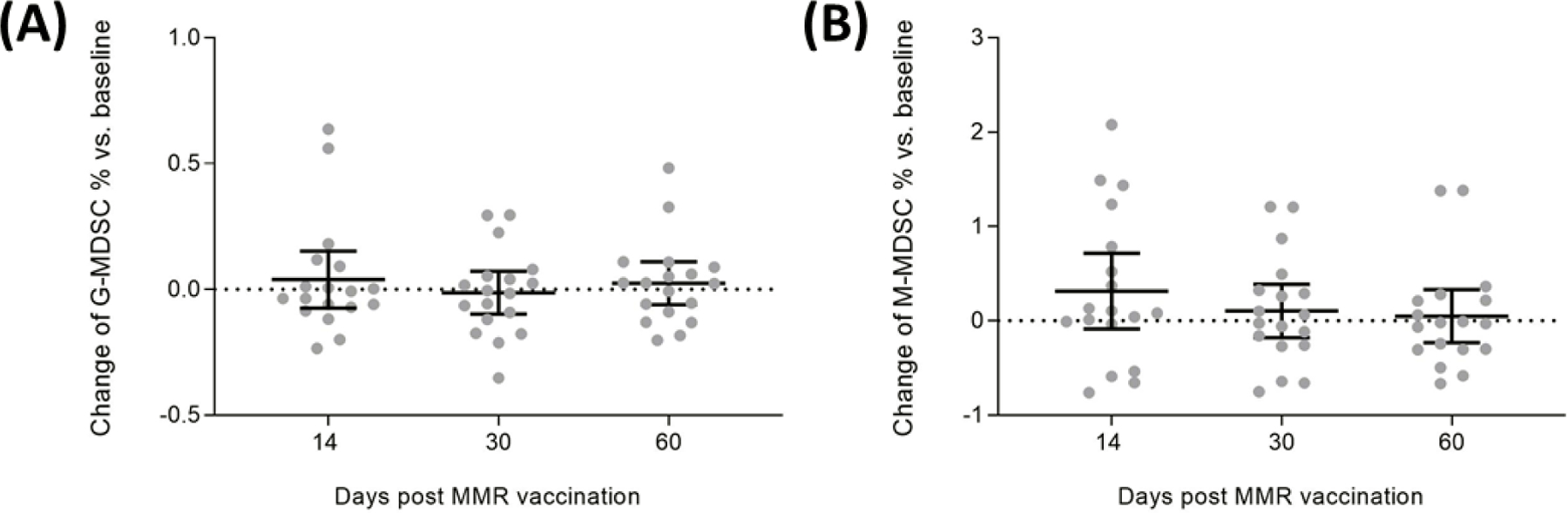
Figure 1. Changes of circulating myeloid-derived suppressor cells at 14-, 30-, and 60-day post-MMR vaccination for the New Orleans cohort Changes of circulating MDSCs at 14-, 30-, and 60-day post-MMR vaccination for the New Orleans (LSUHSC) cohort. Enrolled participants were given either the MMR vaccine or placebo with blood sampling done at baseline and 14, 30, and 60 days post-injection. Figure shows the results of the MMR group (n=18). A. G-MDSC subset. B. M-MDSC subset. The bars indicate means and 95% confidence intervals. There were no significant changes between each subsequent visit and baseline (all p-values>0.05).

The study enrolled 34 participants although only 33 were included in the analysis (15 in the placebo group and 18 in the MMR group) for the analysis (one subject voluntarily withdrew). Differences in percentages of MDSC populations in blood leukocytes between baseline and the subsequent time points post-MMR vaccination for the MMR group are shown in Figure 1. No significant differences in the percentages of M-MDSC or G-MDSC were detected at 14, 30 days, or 60 days post-MMR vaccination. The detailed results for percentage changes in G-MDSC and M-MDSC subsets are shown in Table S1.

**Figure 2.**
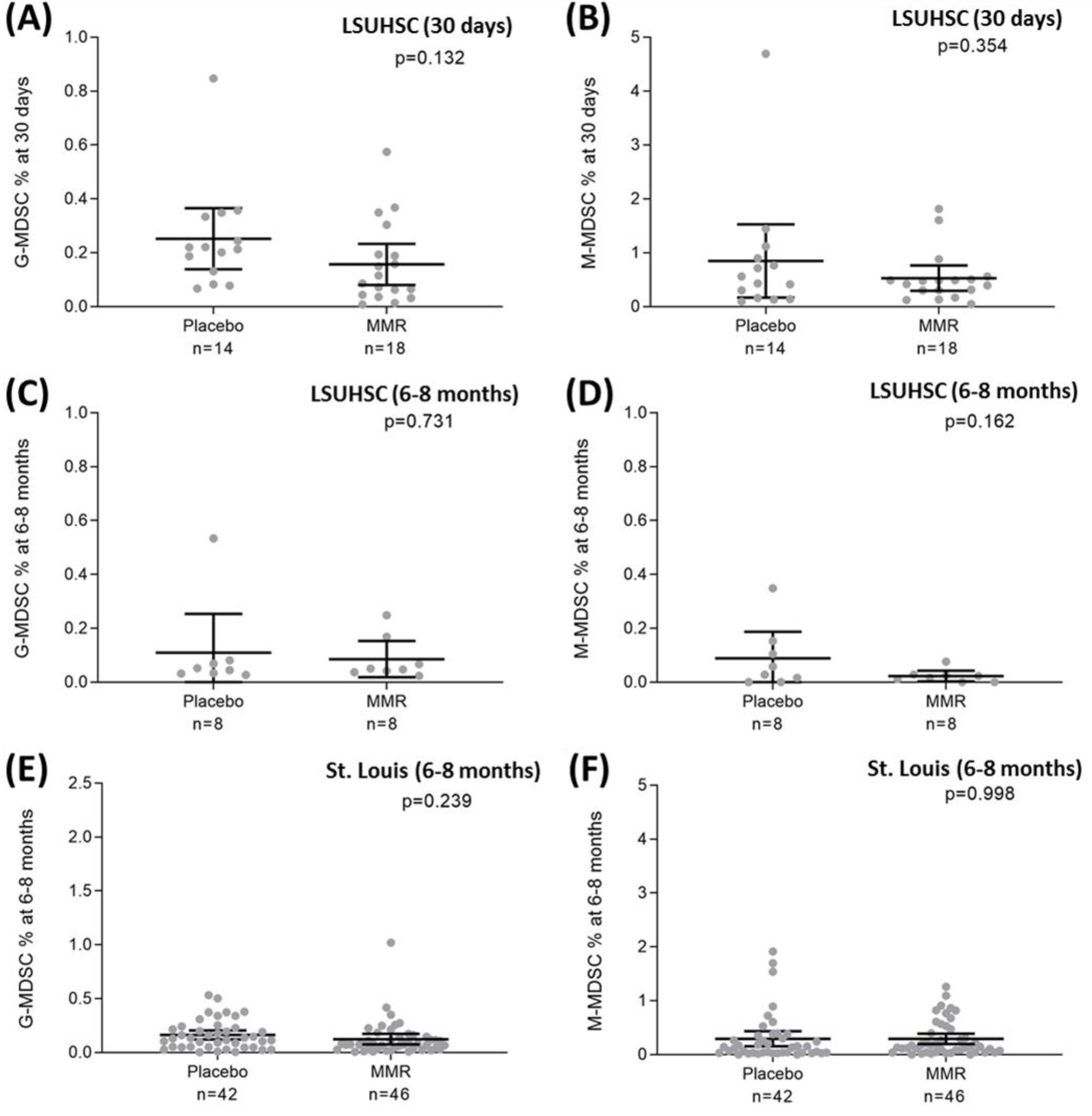
Distributions of circulating myeloid-derived suppressor cells at 30-day post-injection for the New Orleans cohort and 6-8-month post-injection for the New Orleans cohort and the St. Louis cohort Distributions of circulating MDSCs at 30-day post-injection for the New Orleans (LSUHSC) cohort and 6–8-month post-injection for the New Orleans cohort and the St. Louis cohort. Enrolled participants were given either the MMR vaccine or placebo with blood sampling done at baseline and 14, 30, and 60 days post-injection. Figure shows the results for 30-day post-injection for both the MMR vaccine and placebo groups of the New Orleans cohort, and 6–8 months post-injection for the MMR vaccine and placebo groups of the New Orleans cohort and the St. Louis cohort. The bars indicate means and 95% confidence intervals. The p-values were based on Student’s t-tests.

Baseline percentages of M-MDSCs and G-MDSCs in blood leukocytes were similar for the placebo and MMR groups (M-MDSC: 0.34% and 0.43%, respectively, p=0.387; G-MDSC: 0.26% and 0.18%, respectively, p=0.238) (Table S2). In addition to comparisons to baseline MDSC levels (Fig. 1), a separate analysis was conducted comparing the percentages of the two MDSC subsets in those administered the placebo versus MMR vaccination for concurrent times post-injection. Together with the LSUHSC cohort, samples were also included from the St. Louis, MO cohort. Analyses included comparisons between placebo and MMR-vaccinated at 30-day post-vaccination for the LSUHSC cohort and at 6–8 months post-vaccination for both the LSUHSC and St. Louis, MO cohorts. As shown in Figure 2 there were no significant differences detected in the two MDSC subsets between those given the placebo or MMR 30 days (Figures 2A, B) or 6–8 months (Figures 2C, D) post-injection in the LSUHSC cohort, and 6–8 months post-injection in the St. Louis cohort (Figures 2E, F). The detailed results of M-MDSC and G-MDSC percentages by study group and time points are shown in Table S2.

There was one case of COVID-19 in the New Orleans cohort. The case was reported 10 months post-baseline sampling and was in the MMR group. The subject had also received a COVID-19 vaccine 11 months previously with subsequent boosters. The infection occurred during the Omicron variant outbreak (winter 2021) and symptoms reported were very mild, with only some fatigue and lethargy. Samples were taken during the infection period despite the mild symptoms. The percentage of MDSCs remained unchanged (M-MDSC – baseline 0.15% vs COVID+ 0.10%; G-MDSC – baseline 0.024% vs COVID+ 0.022%). The positive case was confirmed by SARS-CoV-2 PCR and serum antibodies for the SARS-CoV-2 nucleocapsid. No other subjects were positive for SARS-CoV-2 via PCR or serum nucleocapsid antibodies.

**Figure 3.**
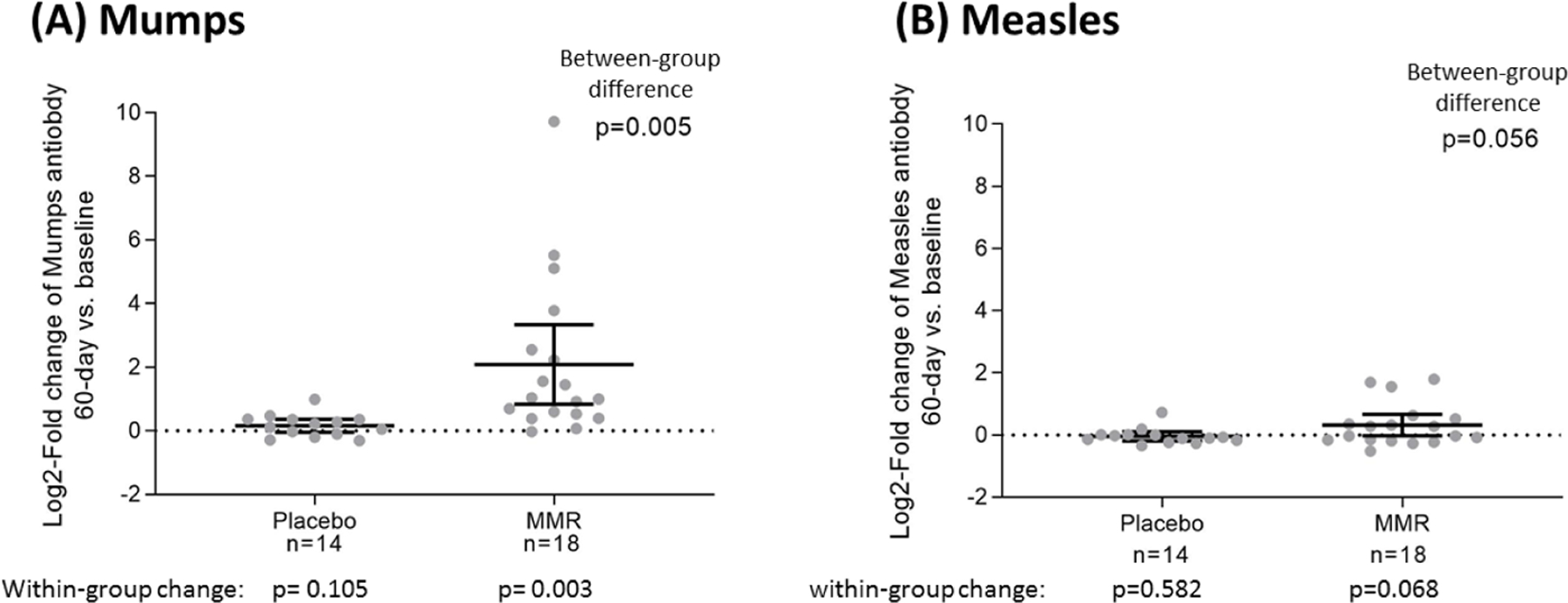
Mumps and Measles serology 60-days post-injection for the placebo and MMR groups Mumps and measles serology 60-day post-injection for the placebo and MMR groups. Enrolled participants in the New Orleans cohort were given either the MMR vaccine or placebo with blood sampling done at baseline and 14, 30, and 60 days post-injection. Figure shows the mumps and measles serology at 60-days post-injection. The bars indicate means and 95% confidence intervals. The p-values of testing between-group differences were based on Student’s t-tests. The p-values of testing within-group changes of 60-day post-MMR vaccination and baseline were based on paired Student’s t-tests.

MMR-associated serology was also evaluated at day 60 post-MMR vaccination or placebo injection compared to baseline for the New Orleans cohort. Results show that antibodies to both measles and mumps virus were increased in those administered the MMR vaccine, whereas no increases were seen in those that received the placebo injection. Antibodies to mumps virus were significantly increased at 60-days post-MMR vaccination compared to baseline (p=0.003). There was a trend for increased antibody titers to measles at 60-days post-MMR vaccination with a marginal significance (p=0.068). Group comparisons in mumps and measles virus antibody titers were also evaluated at 60-days post-injection (Figure 3). For mumps antibodies, those in the MMR group had significantly elevated levels compared with those in the placebo group (p=0.005). For measles antibodies, a similar trend toward higher levels in the MMR group was evident (p=0.056). The overall rate of positive antibody response to the MMR vaccine based on measles and mumps serology was ~a70%.

**Figure 4.**
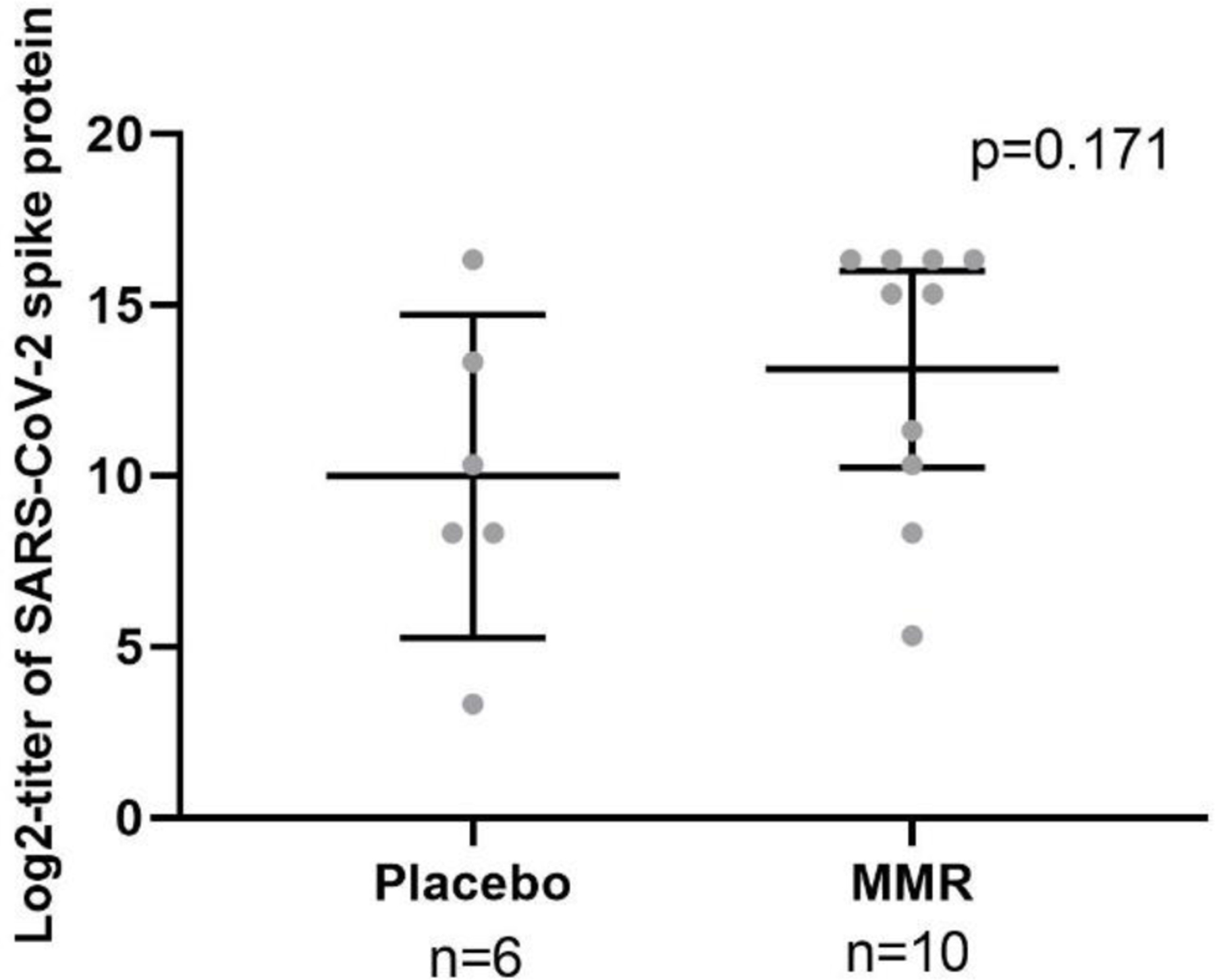
SARS-CoV-2 spike protein titers post COVID-19 vaccination in those given the MMR vaccine or placebo injection SARS-CoV-2 spike protein titers post COVID-19 vaccination in those given the MMR vaccine or placebo injection. Enrolled participants in the New Orleans cohort were given either the MMR vaccine or placebo with blood sampling done at baseline and 14, 30, and 60 days post-injection. All participants were given one of the COVID-19 vaccines before or during enrollment in the study. Figure show SARS-CoV-2 spike protein antibody titers > 2 months post-COVID-19 vaccination using blood samples post-MMR or placebo injection. The bars indicate means and 95% confidence intervals. The p-values were based on Student’s t-test.

We also evaluated whether individuals administered the MMR vaccine developed a stronger antibody response to the subsequent administration of one of the mRNA COVID-19 vaccines (Pfizer or Moderna). For this, sixteen participants from the New Orleans cohort who were COVID-19 vaccinated after enrollment into the trial (n=10 in the MMR group; n=6 in the placebo group) were evaluated for differences in SARS-CoV-2 spike protein antibody titers using log2-transformed titer levels in serum. Serum samples included any/all study-associated sample collections >2 months post-COVID-19 vaccination. While there was a trend toward increased titers of SARS-CoV-2 spike protein antibody in the MMR group compared with the placebo group, this difference was not statistically significant (p=0.171) (Figure 4). This trend was supported by a higher percentage of those in the MMR group having high (Log2-transformed titers >15.3 or raw titers >40,000) SARS-CoV-2 spike protein antibody titers compared to those in the placebo group (60% vs. 17%, respectively, but this was also not statistically significant (p=0.145).

## Discussion

The lack of demonstrable changes in MDSC levels post-MMR vaccination were not totally unexpected, including early or later time points. As noted previously the MDSC populations comprise <1% of leukocytes in the blood. While changes in either M-MDSC or G-MDSC subsets were hypothesized for those administered the MMR vaccine, showing detectable differences would have required considerable expansion in study enrollment and/or minimal variability between individuals. Unfortunately, changes post-vaccination were relatively small with significant variability. Hence, the study lacked the statistical power to identify clear differences. Secondly, although blood is the only compartment that can be evaluated in a clinical setting for such cells, the current hypothesis is that LAV-induced MDSCs reside primarily in the bone marrow until recruited in response to an infectious insult (reviewed in ^10^). Under this predication large numbers of MMR-induced MDSCs would not be detected in blood until they were signaled to migrate out of the bone marrow by an infection such as SARS-CoV-2-related septic event. As no COVID-19-related sepsis cases occurred during the trial, likely mitigated in part by emergency authorization of novel COVID-19 vaccines for HCWs, there was no instance where MDSCs would have migrated from the bone marrow to be detectable in blood. The only breakthrough case of COVID-19 occurred late in the study, was likely caused by the omicron variant, and only resulted in mild symptoms.

Independent of these clinical data, there remains strong corroborating circumstantial clinical evidence that recipients of the MMR vaccine just prior to or during the pandemic were largely spared from severe lung inflammation and sepsis associated with COVID-19 infection. Anecdotal evidence includes a report from the U.S.S Roosevelt which documented an outbreak of COVID-19 in 955 sailors early in the pandemic, with most reporting minor symptoms and only one hospitalization. The lack of severe COVID associated outcomes may be due to the fact that MMR vaccinations are administered to all U.S. Navy recruits (article link). In addition, epidemiological data demonstrated a correlation between recent administration of the live attenuated measles-rubella vaccine (geographical locations with measles outbreaks) and low COVID-19 death rates compared to reported rates elsewhere within the same time frame.^21^ Interestingly, a recent paper detailed findings from an MMR vaccination campaign following 255 subjects in March 2020 (non-RCT, initiated by a measles outbreak in Mexico), many whom were family members of COVID-19+ subjects. Results showed a 9–14% SARS-CoV-2 infection rate and very mild COVID-19-related symptoms in the subjects that had received the MMR vaccine.^22^ While these data are individually circumstantial, collectively, they strongly support potential beneficial effects of the MMR vaccine against severe symptoms associated with the unrelated SARS-CoV-2 infection.

Another possible example of a non-specific beneficial immune effect of the MMR vaccine can be garnered from our trial. While the sample size was small (n=16), more of the individuals previously vaccinated with MMR had a stronger antibody response (high titers) to the COVID-19 vaccine-induced spike protein compared to those given the placebo (60 vs 17%, respectively). While the non-specific beneficial effects of LAVs have been related primarily to the prevalence or susceptibility to infection, the concept of LAVs enhancing responses to other vaccines is novel. Although we were not able to show statistical significance for an MMR-associated enhanced response to the COVID-19 vaccines, the trend toward a stronger response would support this novel concept.

We contend that the positive effects that have been reported for the MMR vaccine preventing severe COVID-19 disease is due in part to induction of a novel form of trained innate immunity. In this context, live attenuated vaccine microbes train MDSC precursors that function to suppress the cytokine storm that is associated with severe lung inflammation and sepsis.^10^ The original impetus for this concept came from our recently published studies using an animal model of polymicrobial sepsis.^6–9^ We discovered that systemic immunization with a live attenuated fungal strain (*Candida dubliniensis*) or abiotic cell wall products could protect mice against lethal sepsis (intravenous *C. albicans* or intra-abdominal infection with *C. albicans* and *Staphylococcus aureus*).^6–9^ In these models of infection, unimmunized animals succumb to sepsis in 24–48 h while immunized animals exhibit very low sepsis scoring and eventually clear the infection with little to no mortality. Accordingly, these studies demonstrated that protection was associated with reduction in systemic proinflammatory cytokines. ^6–9^ Subsequent mechanistic studies identified Gr-1+ MDSCs as critical for protection and suppression of systemic cytokines^6–9^. In addition, the ability of the live attenuated fungal strain to induce protection correlated with the infiltration of the fungi into the bone marrow.^6^ Therefore, we hypothesized that interaction of the attenuated fungal strain with MDSC precursors in the bone marrow leads to training via epigenetic reprogramming. These trained MDSCs can be then activated and released upon exposure to a lethal challenge and exert suppressive effects to ablate sepsis as a novel form of trained innate immunity called ‘trained tolerogenic immunity’.^10^ Recognizing that severe COVID-19 cases often result in sepsis, together with circumstantial data supporting the positive effects of a recent vaccination with a live attenuated vaccine against severe COVID-19, we proposed this concept for mitigation of COVID-19 sequelae. This ultimately led to the subsequent initiation of the MMR clinical trial.^15^

Several other groups also proposed or initiated clinical trials with MMR or other LAVs recognizing the clinical observations being reported.^17,19,23–26^ However, the proposed mechanisms for protection induced by the LAVs included enhanced trained innate immunity via cytokine production such as type I interferon, or alternatively, cross-reactivity of SARS-CoV-2 virus to antibodies generated in response to the respective vaccine. This also prompted several retrospective studies evaluating correlations of prior MMR vaccinations with COVID-19-related symptoms or molecular analyses of antibodies specific to measles, mumps, or rubella for cross-reactivity to SARS-CoV-2 ^24,27–30^. As for prospective randomized controlled trials (RCT), in Brazil, a RCT comparing MMR vs. placebo was largely completed prior to distribution of COVID-19 vaccine. Results showed that those receiving the MMR vaccine exhibited ~50% risk reduction in COVID symptoms following infection and >75% reduction in COVID-related treatment regimens.^20^ In Europe, a double-blinded RCT comparing BCG vaccination vs. placebo in individuals at risk for COVID-19 was initiated prior to distribution of COVID vaccines.^19^ The trial included volunteers >50 yrs of age and results showed that BCG-vaccinated individuals had a 68% reduction of risk to develop COVID-19 in a 6-month follow-up. Of the eight participants hospitalized for SARS-CoV-2 infection, two were in the BCG group and 6 in the placebo group. While the sample size was small in many of the studies, together they continue to support the beneficial non-specific immune effects of LAVs for unrelated infections, either by tolerogenic TII, enhanced TII, or some level of cross-reactivity. The CROWN CORONATION study is still under analysis with results pending.

There were several challenges during the trial that were mostly related to enrollment. First, COVID vaccines became available shortly after the trial began and was distributed first to HCW. This affected both the LSUHSC cohort and the St. Louis cohort and effectively compromised the ability to use COVID health status as an outcome. In the case of the LSUHSC cohort, which had slower recruitment than the St. Louis cohort, interest in the trial waned as the COVID-19 vaccines became available. In response to this, enrollment was quickly extended to the general public who had not yet received one of the COVID-19 vaccines. However, attempts to enroll large numbers were relatively unsuccessful prior to the expanded emergency authorization of COVID-19 vaccines for the general public. We also noted that up to 50% of HCW staff in area nursing homes were reluctant to accept COVID-19 vaccines. However, they were also reluctant to participate in the MMR clinical trial, likely due to widespread misinformation and vaccine hesitancy. Other incentives were offered including off-site enrollment and compensation for time/transportation but these also failed to stimulate additional interest. Finally, prior to the funding of the trial a grass roots campaign for adult MMR vaccination had been established based on all the circumstantial evidence surrounding the positive effects of LAVs. Hence, many potential trial participants opted to get the MMR vaccine at their local pharmacy rather than enroll in the trial and risk a placebo randomization.

Despite the inconclusive results for this MMR-COVID trial, the evidence remains strong for LAVs such as MMR to play a role in mitigating severe inflammation and sepsis. As additional clinical trials are initiated potentially to continue to test the hypothesis, we provide several important lessons learned from this trial that can be applied to future trials. First, since blood is the lone minimally-invasive compartment to monitor changes in MDSCs, and with percentages of MDSCs in the blood extremely low, the primary outcome for future trials should be health status that includes monitoring sepsis scoring and powered adequately for prevalence of sepsis in any given population. The MDSC evaluation should be a secondary outcome with expected increases in blood during recovery from a sepsis diagnosis. Therefore, there is no need to conduct multiple blood samplings other than baseline and potentially one interim sample for LAV (MMR) response verification. Relative to the MMR vaccine responses, our results showed a ~70% response rate in a population with a median age of ~50 yrs, Moreover, we were only able to show a clear significant increase in antibody titers to the mumps virus post-MMR vaccination compared to baseline with a trend toward an increase in antibody titers to measles virus. Hence, we recommend that a booster be given at ~30 days post-initial MMR vaccination for a stronger response. Finally, for optimal participant enrollment it is recommended to incorporate the simplest design possible without compromising the results. Because the participants for this type of trial are all healthy volunteers rather than patients with a disease/syndrome-associated (common to most clinical trials), making it as easy as possible to participate will increase probability of strong enrollment. While randomized control trials are preferred, use of an experimental design that does not include a formal placebo group (e.g. quasi-experimental) may still provide informative data. An example would be testing in a geriatric population that may not consent to several blood draws, especially if randomized to the control group with little to no benefit. This does limit the blinding of the trial but likely would not compromise the results of gathering data on sepsis diagnoses in the entire population.

## Conclusion

In conclusion, while these current trials were largely inconclusive for the outcomes expected, this should not deter future trials to test the hypothesis that LAVs induce/active MDSCs that can mitigate severe inflammation/sepsis. To this end, future trials incorporating suggestions from this trial may include nursing home residents where sepsis can be common and lead to significant mortality. Currently there are no vaccines or preventive therapies for sepsis of broad microbial origin. Testing the effects of the MMR to reduce the prevalence of lethal sepsis, along with evidence for increases in peripheral MDSCs following recovery from confirmed cases, may have a significant impact on the incidence of sepsis nationwide or worldwide.

## Supplementary Material

1

## References

[1] Aaby P, Benn CS. Developing the concept of beneficial non-specific effect of live vaccines with epidemiological studies. Clin Microbiol Infect Dec 2019;25(12):1459–1467. doi:10.1016/j.cmi.2019.08.011 31449870

[2] Gyssens IC, Netea MG. Heterologous effects of vaccination and trained immunity. Clin Microbiol Infect Dec 2019;25(12):1457–1458. doi:10.1016/j.cmi.2019.05.024 31158520

[3] Moorlag S, Arts RJW, van Crevel R, Netea MG. Non-specific effects of BCG vaccine on viral infections. Clin Microbiol Infect Dec 2019;25(12):1473–1478. doi:10.1016/j.cmi.2019.04.020 31055165

[4] Sinzinger AX, Von Kries R, Siedler A, Wichmann O, Harder T. Non-specific effects of MMR vaccines on infectious disease related hospitalizations during the second year of life in high-income countries: a systematic review and meta-analysis. Hum Vaccin Immunother Mar 3 2020;16(3):490–498. doi:10.1080/21645515.2019.1663119 31625797 PMC7227673

[5] Kaufmann E, Sanz J, Dunn JL, BCG Educates Hematopoietic Stem Cells to Generate Protective Innate Immunity against Tuberculosis. Cell Jan 2018;172(1–2):176–190.e19. doi:10.1016/j.cell.2017.12.031 29328912

[6] Lilly EA, Yano J, Esher SK, Hardie E, Fidel PL Jr., Noverr MC. Spectrum of Trained Innate Immunity Induced by Low-Virulence Candida Species against Lethal Polymicrobial Intra-abdominal Infection. Research Support, N.I.H., Extramural. Infect Immun Aug 2019;87(8)doi:10.1128/IAI.00348-19 PMC6652762 31085710

[7] Lilly EA, Ikeh M, Nash EE, Fidel PL Jr., Noverr MC. Immune Protection against Lethal Fungal-Bacterial Intra-Abdominal Infections. Research Support, N.I.H., Extramural. mBio Jan 16 2018;9(1)doi:10.1128/mBio.01472-17 PMC5770546 29339423

[8] Lilly EA, Bender BE, Esher Righi S, Fidel PL Jr., Noverr MC. Trained Innate Immunity Induced by Vaccination with Low-Virulence Candida Species Mediates Protection against Several Forms of Fungal Sepsis via Ly6G(+) Gr-1(+) Leukocytes. Research Support, N.I.H., Extramural. mBio Oct 26 2021;12(5):e0254821. doi:10.1128/mBio.02548-21 34663098 PMC8524338

[9] Harriett AJ, Esher Righi S, Lilly EA, Fidel P Jr., Noverr MC. Efficacy of Candida dubliniensis and Fungal beta-Glucans in Inducing Trained Innate Immune Protection Against Inducers of Sepsis. Research Support, N.I.H., Extramural. Front Cell Infect Microbiol 2022;12:898030. doi:10.3389/fcimb.2022.898030 35770067 PMC9234138

[10] Esher SK, Fidel PL Jr., Noverr MC. Candida/Staphylococcal Polymicrobial Intra-Abdominal Infection: Pathogenesis and Perspectives for a Novel Form of Trained Innate Immunity. Review. J Fungi (Basel) May 9 2019;5(2)doi:10.3390/jof5020037 PMC6617080 31075836

[11] Quintin J, Saeed S, Martens JHA, Candida albicans infection affords protection against reinfection via functional reprogramming of monocytes. Research Support, N.I.H., Extramural Research Support, Non-U.S. Gov’t. Cell Host Microbe Aug 16 2012;12(2):223–32. doi:10.1016/j.chom.2012.06.006 22901542 PMC3864037

[12] Kleinnijenhuis J, Quintin J, Preijers F, Bacille Calmette-Guerin induces NOD2-dependent nonspecific protection from reinfection via epigenetic reprogramming of monocytes. Research Support, N.I.H., Extramural Research Support, Non-U.S. Gov’t. Proc Natl Acad Sci U S A Oct 23 2012;109(43):17537–42. doi:10.1073/pnas.1202870109 22988082 PMC3491454

[13] Saeed S, Quintin J, Kerstens HH, Epigenetic programming of monocyte-to-macrophage differentiation and trained innate immunity. Research Support, Non-U.S. Gov’t. Science Sep 26 2014;345(6204):1251086. doi:10.1126/science.1251086 25258085 PMC4242194

[14] Zhou F, Yu T, Du R, Clinical course and risk factors for mortality of adult inpatients with COVID-19 in Wuhan, China: a retrospective cohort study. Lancet Mar 28 2020;395(10229):1054–1062. doi:10.1016/S0140-6736(20)30566-3 32171076 PMC7270627

[15] Fidel PL Jr., Noverr MC. Could an Unrelated Live Attenuated Vaccine Serve as a Preventive Measure To Dampen Septic Inflammation Associated with COVID-19 Infection? Research Support, N.I.H., Extramural. mBio Jun 19 2020;11(3)doi:10.1128/mBio.00907-20 PMC7304316 32561657

[16] Ld Toit, Gupta A Dehbi H-M, THE EFFECT OF THE MEASLES, MUMPS AND RUBELLA VACCINE ON INNATE AND ADAPTIVE IMMUNE RESPONSES IN PERSONS RECEIVING A SARS-COV-2 mRNA VACCINE. medRxiv 2022:2022.09.09.22279771. doi:10.1101/2022.09.09.22279771

[17] Giamarellos-Bourboulis EJ, Tsilika M, Moorlag S, Activate: Randomized Clinical Trial of BCG Vaccination against Infection in the Elderly. Cell Oct 15 2020;183(2):315–323 e9. doi:10.1016/j.cell.2020.08.051 32941801 PMC7462457

[18] O’Connor E, Teh J, Kamat AM, Lawrentschuk N. Bacillus Calmette Guerin (BCG) vaccination use in the fight against COVID-19 - what’s old is new again? Editorial. Future Oncol Jul 2020;16(19):1323–1325. doi:10.2217/fon-2020-0381 32406253 PMC7222530

[19] Tsilika M, Taks E, Dolianitis K, ACTIVATE-2: A Double-Blind Randomized Trial of BCG Vaccination Against COVID-19 in Individuals at Risk. Multicenter Study Randomized Controlled Trial Research Support, Non-U.S. Gov’t. Frontiers in immunology 2022;13:873067. doi:10.3389/fimmu.2022.873067 35865520 PMC9294453

[20] Fedrizzi EN, Girondi JBR, Sakae TM, EFFICACY OF THE MEASLES-MUMPS-RUBELLA (MMR) VACCINE IN THE REDUCING THE SEVERITY OF COVID-19: AN INTERIM ANALYSIS OF A RANDOMISED CONTROLLED CLINICAL TRIAL. medRxiv 2021:2021.09.14.21263598. doi:10.1101/2021.09.14.21263598

[21] Gold J MMR Vaccine Appears to Confer Strong Protection from COVID-19: Few Deaths from SARS-CoV-2 in Highly Vaccinated Populations 2020.

[22] Larenas-Linnemann DE, Rodriguez-Monroy F. Thirty-six COVID-19 cases preventively vaccinated with mumps-measles-rubella vaccine: All mild course. Letter. Allergy Sep 7 2020;doi:10.1111/all.14584 32894782

[23] Sharma D Repurposing of the childhood vaccines: could we train the immune system against the SARS-CoV-2. Expert review of vaccines Sep 2021;20(9):1051–1057. doi:10.1080/14760584.2021.1960161 34313516 PMC8425442

[24] Lundberg L, Bygdell M, Stukat von Feilitzen G, Recent MMR vaccination in health care workers and Covid-19: A test negative case-control study. Research Support, Non-U.S. Gov’t. Vaccine Jul 22 2021;39(32):4414–4418. doi:10.1016/j.vaccine.2021.06.045 34187707 PMC8216866

[25] Anbarasu A, Ramaiah S, Livingstone P. Vaccine repurposing approach for preventing COVID 19: can MMR vaccines reduce morbidity and mortality? Research Support, Non-U.S. Gov’t. Hum Vaccin Immunother Sep 1 2020;16(9):2217–2218. doi:10.1080/21645515.2020.1773141 32501133 PMC7553692

[26] Ashford JW, Gold JE, Huenergardt MA, MMR Vaccination: A Potential Strategy to Reduce Severity and Mortality of COVID-19 Illness. Editorial. The American journal of medicine Feb 2021;134(2):153–155. doi:10.1016/j.amjmed.2020.10.003 33198951 PMC7583585

[27] Gold JE, Baumgartl WH, Okyay RA, Analysis of Measles-Mumps-Rubella (MMR) Titers of Recovered COVID-19 Patients. mBio Nov 20 2020;11(6)doi:10.1128/mBio.02628-20 PMC7686805 33219096

[28] Hassani D, Amiri MM, Maghsood F, Does prior immunization with measles, mumps, and rubella vaccines contribute to the antibody response to COVID-19 antigens? Iranian journal of immunology : IJI Mar 2021;18(1):47–53. doi:10.22034/iji.2021.87990.1843 33787513

[29] Yengil E, Onlen Y, Ozer C, Hambolat M, Ozdogan M. Effectiveness of Booster Measles-Mumps-Rubella Vaccination in Lower COVID-19 Infection Rates: A Retrospective Cohort Study in Turkish Adults. International journal of general medicine 2021;14:1757–1762. doi:10.2147/IJGM.S309022 33994804 PMC8113608

[30] Young A, Neumann B, Mendez RF, Homologous protein domains in SARS-CoV-2 and measles, mumps and rubella viruses: preliminary evidence that MMR vaccine might provide protection against COVID-19. medRxiv 2020:2020.04.10.20053207. doi:10.1101/2020.04.10.20053207

