# Supplementary material for: Effect of MMR Vaccination to Mitigate Severe Sequelae Associated With COVID-19: Challenges and Lessons Learned
Source: Med Res Arch. Author manuscript; Available in PMC 2023 May 5. (PMC10162774; doi:10.18103/mra.v11i2.3598)
Supplement: 1 [file NIHMS1890114-supplement-1.pdf]

SUPPLEMENTARY MATERIAL

Supplemental Table 1. Changes of circulating Myeloid-derived suppressor cells at 14-, 30-, 60-day, and 6-8 months post-MMR vaccination for the New Orleans cohort

Changes of MDSC between 2 visits	G-MDSC Placebo	MMR		M-MDSC Placebo	MMR	
	mean± SD (n)[1]	mean± SD (n)[1]	p-value[2]	mean± SD (n)[1]	mean± SD (n)[1]	p-value[2]
14 vs. 0 days	-	0.04± 0.23 (18)	-		0.32± 0.80 (18)	-
30 vs. 0 days	-0.03± 0.15 (14)	-0.01± 0.17 (18)	0.805	0.49± 1.01 (14)	0.11± 0.57 (18)	0.179
60 vs. 0 days	-	0.02± 0.17 (18)	-		0.05± 0.57 (18)	-
6-8 m vs. 0 days	-0.07± 0.17 (8)	-0.06± 0.09 (8)	0.790	-0.32± 0.33 (8)*	-0.46± 0.30 (8)*	0.373

[1]mean± standard deviation (SD). For within-group comparison, there was no significant (p-value>0.05) changes between the subsequent visits and baseline for granulocytic-MDSC (G-MDSC) and Monocytic-MDSC (M-MDSC) under most of the conditions based on the paired Student's t-test, except the M-MDSC changes between 6-8 months post MMR vaccination and baseline: p=0.03 for placebo and p=0.004 for MMR
[2]based on the the Student's t-test.

Supplemental Table 2. Granulocytic myeloid-derived suppressor cells (G-MDSC) and monocytic myeloid-derived suppressor cells (M-MDSC) by study group and time-points

	G-MDSC			M-MDSC		
	Placebo mean± SD (n)[1]	MMR mean± SD (n)[1]	p-value[1]	Placebo mean± SD (n)[1]	MMR mean± SD (n)[1]	p-value[1]
**New Orleans**						
Baseline	0.26± 0.26 (15)	0.18± 0.12 (19)	0.238	0.34±0.32 (n=15)	0.43± 0.28 (19)	0.387
14 days	-	0.21± 0.24 (18)	-	-	0.74± 0.78 (18)	-
30 days	0.25± 0.20 (14)	0.16± 0.15 (18)	0.132	0.85± 1.18 (n=14)	0.53± 0.47 (18)	0.354
60 days	-	0.19± 0.19 (18)	-	-	0.47± 0.50 (18)	-
6-8 months	0.11± 0.17 (8)	0.09± 0.08 (8)	0.731	0.09± 0.12 (8)	0.02± 0.02 (8)	0.162
**St. Louis**						
6-8 months	0.16± 0.14 (42)	0.13± 0.16 (46)	0.239	0.29± 0.45 (42)	0.29± 0.33 (46)	0.998

[1] mean± standard deviation (sample size), based on the Student's t-test
